# Xanthogranuloma of the external auditory canal—an atypical anatomical manifestation

**DOI:** 10.1093/jscr/rjaa536

**Published:** 2021-03-31

**Authors:** James T Connell, Thu Nguyen, Andrew S Carney, Sheldon Chong

**Affiliations:** Department of Otolaryngology Head and Neck Surgery, Modbury Hospital, Adelaide, South Australia, Australia; Adelaide Medical School, Faculty of Health and Medical Sciences, University of Adelaide, Adelaide, South Australia; Adelaide Medical School, Faculty of Health and Medical Sciences, University of Adelaide, Adelaide, South Australia; College of Medicine and Public Health, Flinders University, Adelaide, South Australia, Australia; Department of Otolaryngology Head and Neck Surgery, Modbury Hospital, Adelaide, South Australia, Australia; Adelaide Medical School, Faculty of Health and Medical Sciences, University of Adelaide, Adelaide, South Australia

## Abstract

Juvenile xanthogranuloma is a proliferative cutaneous manifestation encountered in the paediatric population. Adult cases are uncommon, but have been reported. Lesions are prevalent in the head and neck region, but rarely observed in the external auditory canal. We present the case of a 39-year-old female with a rapidly progressing obstructive soft tissue lesion of the external auditory canal. Surgical excision diagnosed the lesion as a rarely observed otological manifestation of juvenile xanthogranuloma. Surgical excision was curative with no locoregional recurrence. Otolaryngologists should consider juvenile xanthogranuloma as a differential for atypical soft tissue cutaneous lesions of the head and neck, including in divergent populations.

## INTRODUCTION

Juvenile xanthogranuloma (JXG) is a proliferative cutaneous disorder of infancy most commonly encountered in the first year of life, although adult cases have been previously reported [1]. Lesions follow an indolent course, manifesting as solitary or clustered papules that recede over months to years. Anatomical distribution is characteristically the head, neck and trunk. Incidence in the external ear is extremely rare with very few published case reports [1–4]. Although lesions are classically benign, their presence in atypical anatomical sites in divergent population groups can provoke suspicion for more sinister differentials diagnoses.

## CASE PRESENTATION

**Figure 1:**
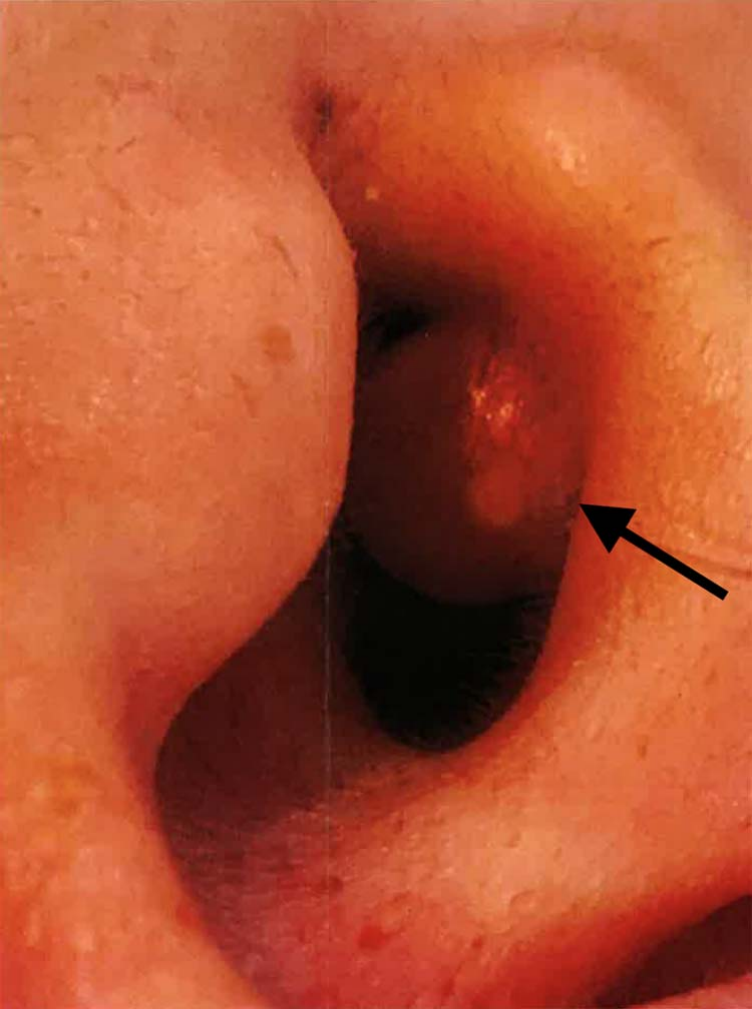
Clinical photograph of the juvenile xanthogranuloma (arrow) obstructing the left external auditory canal.

A 39-year-old female was referred to our department with a red fleshy lesion occupying her left external auditory canal. The lesion developed insidiously and grew rapidly over the course of months. On clinical evaluation, a well circumscribed, epithelialized lesion was identified deep to the conchal bowl within the external auditory meatus. It was broadly pedicled to the antero-superior canal wall. The lesion was 8 mm in diameter with near complete occlusion of the canal (Fig. 1). The texture was soft and elastic, and the complexion was red. Attempts to aspirate the contents with a fine-bore needle were futile.

**Figure 2:**
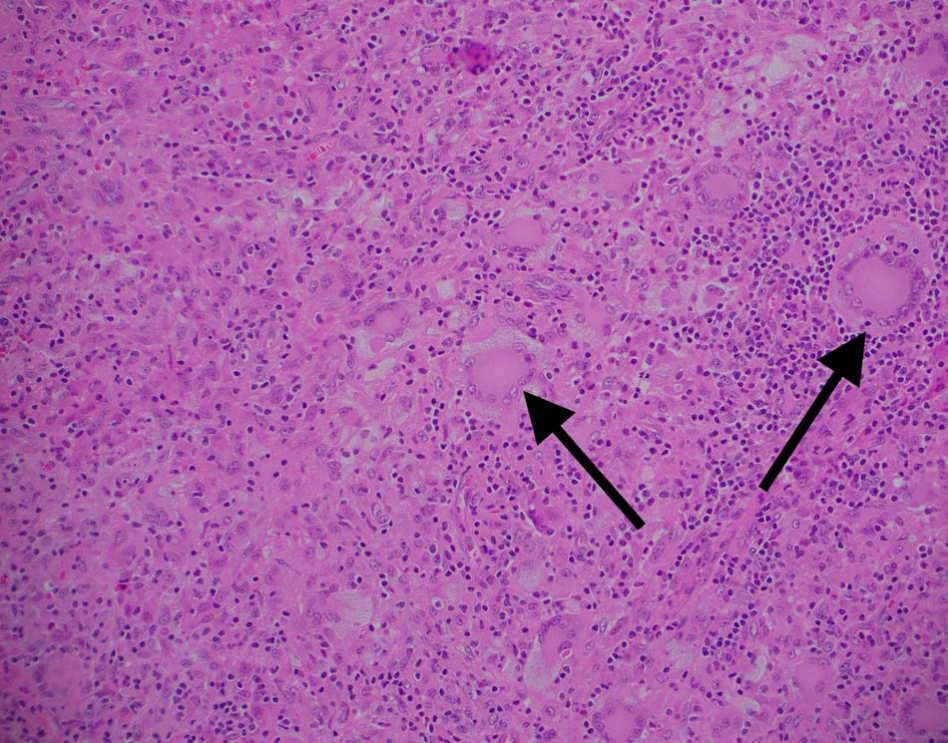
Haematoxylin and eosin stained ×100 magnification histopathology slide from excised xanthogranuloma highlighting characteristic Touton cells (arrow) amongst abundant monocytes.

Given the brisk progression and uncertain aetiology, surgical excision was undertaken. Local infiltration with xylocaine was used for anaesthesia and to aid hydro dissection. Sharp dissection of the broad base with a combination of scalpel and dissecting scissors provided en bloc resection of the lesion. When dividing the tanned, fatty contents from the underlying meatal cartilage, no clearly demarcated tissue plane or capsule could be identified. Haemostasis was achieved with bipolar diathermy and the defect was primarily closed with absorbable sutures. Histological assessment of the 8 × 9 × 6 mm lesion identified diffuse sheets of mononuclear and multinucleated histiocytes within the dermis. Eosinophilia, foamy cytoplasms and Touton cells were described. These findings were characteristic and diagnostic of JXG (Fig. 2). There was no evidence of cellular atypia or dysplasia. The cytological findings extended to the deep positive margin. At the post-operative evaluation, the surgical site had healed well with restoration of anatomy. At long-term follow-up, there was no evidence of local recurrence.

## DISCUSSION

JXG are an uncommon manifestation of non-Langerhans cell histiocytosis. The characteristic microscopic findings are abundant histiocytes, foam cells and Touton cells [4, 5]. The predominant cell types are macrophages and monocytes with an invariable absence of Langerhans cells. Cells stain strongly for the macrophage marker, CD68, and are negative for the SD-100 protein of Langerhans cells [3].

Classic clinical morphology is the insidious development of red papules that can evolve into yellow, scaly nodules in a solitary or clustered configuration. Lesions typically plateau at 5 mm in size, but can progress beyond 20 mm. The natural history in children is to regress completely over months to years. JXG are overwhelmingly more common in the paediatric population with a greater incidence in Caucasians and males [6]. The incidence in adulthood is infrequent, accounting for 10% of cases. Adult variants have a more pervasive pathophysiology and may never regress without definitive intervention.

JXG can occur at any cutaneous site, but the primary distribution is the skin of the head, neck and trunk. Atypical non-cutaneous lesions have been described in the oral cavity, central nervous system, gastrointestinal tract, lungs, bones and kidneys. Lesions occurring in the external auditory canal are incredibly rare. Previous case reports in the ear describe otorrhoea, bleeding and hearing loss [2, 3, 7, 8]. Lesions within the auditory canal have the capacity to cause infection, disrupt desquamation and impede acoustic conduction.

The swift growth and variable morphological features can mimic other benign and malignant pathologies of the external ear. Exostoses, osteoma, adenoma and ceruminoma share common physical traits with JXG [7]. The soft and compressible texture of JXG is comparable with polyps, lipomas or cysts. The rapid progression and crusted epithelial surface in established lesions can provoke suspicious for cholesteatoma or malignancy.

The majority of cases are treated expectantly; however, in an atypical site like the external ear, the potential for local complications coupled with diagnostic uncertainty justifies a more invasive approach. Surgical excision provides accurate diagnosis and symptom resolution. Following surgical resection, JXG rarely recur, irrespective of margins [1].

JXG are a rarely encountered proliferative pathology of the external auditory canal. Although they are invariably benign, the unique anatomical location and spectrum of morphology evokes diagnostic ambiguity. Primary surgical excision provides definitive diagnosis, symptom alleviation and carries a low risk of recurrence. Otolaryngologists should include JXG in their cache of differentials when presented with a rapidly evolving soft tissue lesion of the external ear.

## References

[1] Lee D-H , Kim J. Juvenile xanthogranuloma of the external auditory canal in an adult. Int Adv Otol 2009;5:399–400.

[2] Saleh E , Shaikh A, Jastania R, Zuraqai BA. Juvenile xanthogranuloma of middle ear and external auditory canal: a rare case report. J Otol Rhinol 2017;6. doi: 10.4172/2324-8785.1000313.

[3] Hur J , Kim JK, Kim N, Seo G, Choi W, Byun JS, et al. Adult-onset juvenile xanthogranuloma of the external auditory canal: a case report. J Korean Soc Radiol 2016;74:335.

[4] Misra S , Gupta K, Gupta R. Solitary adult xanthogranuloma in external auditory canal: cyto-histopathological correlation of an uncommon entity at an uncommon site. Diagn Cytopathol 2020;48:666–9. 32275346 10.1002/dc.24430

[5] Püttgen K , Levy M, Corona R. Juvenile Xanthogranuloma (JXG). UpToDate, 2018. https://www.uptodate.com/contents/juvenile-xanthogranuloma-jxg (31 August 2020, date last accessed).

[6] Mrad MA , Chan K, Cypel TK, Zuker RM. Juvenile xanthogranuloma of the ear: a case report. Can J Plast Surg 2008;16:229–31. 19949504 10.1177/229255030801600404 PMC2691030

[7] Yoshihama K , Kato Y, Baba Y. Xanthogranuloma of the external auditory canal mimicking a benign tumor: a case report. Case Rep Otolaryngol 2012;2012:1–3. 10.1155/2012/298089 PMC3420438 22953105

[8] Lin L-H , Lin H-C, Shu M-T, Chen B-F. External auditory canal xanthogranuloma. Otol Neurotol 2013;34:e3. 23187925 10.1097/MAO.0b013e31826938a6

